# Hearing loss characteristics and cerumen management efficacy in low-income South African communities: a cross-sectional study

**DOI:** 10.1017/S1463423625000246

**Published:** 2025-03-10

**Authors:** Sello Marven Manganye, Caitlin Frisby, Tarryn Marisca Reddy, Tersia de Kock, De Wet Swanepoel

**Affiliations:** 1 Department of Speech-Language Pathology and Audiology, University of Pretoria, Pretoria, South Africa; 2 HearX Foundation, Pretoria, South Africa; 3 Virtual Hearing Lab, Collaborative initiative between the University of Colorado and the University of Pretoria, Aurora, CO, USA; 4 Department of Otolaryngology-Head and Neck Surgery, University of Colorado School of Medicine, Aurora, CO, USA

**Keywords:** artificial intelligence, cerumen impaction, cerumen management, community-based care, elderly population, hearing loss, low-income community, task-shifting

## Abstract

**Aim:**

To describe the prevalence and characteristics of hearing loss in a self-referred adult cohort in low-income South African communities and to evaluate the effectiveness of a cerumen management protocol within a community-based service setting.

**Background:**

Hearing loss affects 1.5 billion people globally, with a disproportionate impact on individuals in low- and middle-income countries (LMICs) and the elderly, often attributed to age-related factors and cerumen impaction. Despite the high prevalence, access to ear and hearing care remains challenging, particularly in LMICs, such as Africa.

**Methods:**

A total of 227 participants aged 43–102 were recruited from two community centres in low-income South African communities for hearing evaluation and cerumen management for those with cerumen impaction. A cross-sectional, predominantly quantitative approach was used.

**Findings:**

Video otoscopy of 448 ears revealed normal findings in 57.9%, cerumen impaction in 29.1%, and other abnormalities in 1.3%. The prevalence of confirmed hearing loss was 97.8%, primarily mild (45.8%), and sensorineural hearing loss (SNHL) was the most common (55.3%). Cerumen impaction accompanied hearing loss in 28.4% of cases. Post-treatment, 50.9% of participants with cerumen impaction showed normal otoscopy results, with mean hearing improvements of 16.2 dB (±17.9 SD) in the left ears and 15.8 dB (±17.2 SD) in the right ears, though overall significance was limited.

**Conclusion:**

The high prevalence of hearing loss and cerumen impaction in low-income communities emphasizes the importance of ear care in primary healthcare (PHC) settings, especially for the elderly. Effective community-based cerumen management highlights the potential of integrating community resources and task-shifting strategies for cost-effective ear care in resource-limited settings.

## Background

Globally, hearing loss is the fourth leading cause of disability, impacting over 1.5 billion individuals (Global Burden of Disease, 2021; McDaid *et al.*, 2021; World Health Organization, 2021). Adults aged 60 and above are the most affected, with prevalence rates exceeding 42% (WHO, 2021). Furthermore, age-related hearing loss was reported as the primary cause of global years lived with disability in those older than 70 years (GBD, 2021). The burden is higher in low- and middle-income countries (LMICs), where factors including higher rates of infectious diseases, environmental risks, economic constraints, shortage of hearing healthcare professionals, and limited access to hearing care contribute to elevated prevalence rates (Mulwafu *et al.*, 2016; Cunningham and Tucci, 2017; Mulwafu *et al.*, 2017; Spreckley and Kuper, 2017; Baum *et al.*, 2019; Swanepoel, 2020; GBD, 2021; WHO, 2021; Frisby *et al.*, 2022a; Boisvert *et al.*, 2023). In sub-Saharan Africa, where approximately 15.7% of individuals aged 15 years and older have hearing loss (WHO, 2012; Mulwafu *et al.*, 2016), there is a persistent shortage of research on hearing loss prevalence and causes (Mulwafu *et al.*, 2016; Louw *et al.*, 2018a). The implications of unaddressed hearing loss are profound and multifaceted, influencing participation in daily activities, overall health, well-being, social livelihood, and the economy, particularly in resource-constrained LMICs (Smith *et al.*, 2015; Spreckley and Kuper, 2017; Swanepoel, 2020). Furthermore, approximately $980 billion is lost globally annually due to unaddressed hearing loss (Swanepoel, 2020; McDaid *et al.*, 2021; WHO, 2021). As a result, raising awareness and exploring innovative technologies and service-delivery approaches is an urgent priority (WHO, 2021).

The most common type of hearing loss in adults is sensorineural hearing loss (SNHL), followed by conductive (CHL) and mixed hearing loss (MHL) (Baltussen and Smith, 2012; Liu *et al.*, 2020). However, research on the prevalence of SNHL in Africa is limited (Liu *et al.*, 2020). A Cameroonian population-based review study reported a 61.7–94.4% prevalence of SNHL across different age groups (>15 years) (Fokouo *et al.*, 2015; Tingang *et al.*, 2019; 2020). A study conducted in Egypt reported a 40.7% prevalence of SNHL in adults over 65 years (Abdel-Hamid *et al.*, 2007). Cerumen impaction or excessive cerumen is a common cause of CHL, contributing to 7%–35% of cases across different age groups, particularly affecting adults over 65 years (WHO, 2021; Humes, 2024). Clinically, the amount of cerumen in ear canals varies widely among individuals, even within the same age group, and this variation may or may not directly affect their hearing abilities (Olusanya, 2003; Mulwafu *et al.*, 2016). A systematic review by Mulwafu *et al.* (2016) highlighted that cerumen impaction accounted for 24% of hearing loss in African community- and school-based studies.

Treatment options for hearing loss vary based on the type and severity, with hearing aids most commonly used to treat SNHL and MHL (Müller and Barr-Gillespie, 2015; Brodie *et al.*, 2018; Wardenga *et al.*, 2020). However, less than 2% of individuals with disabling hearing loss in sub-Saharan Africa use hearing aids due to access and affordability issues (Bisgaard *et al.*, 2022). CHL, often transient, may be addressed with medications or surgeries, with cerumen impaction requiring removal (Mulwafu *et al.*, 2016; Hirsch *et al.*, 2017; Vanneste and Page, 2019; Marchioni *et al.*, 2020; WHO, 2021). Cerumen removal may include the use of cerumenolytic agents for softening and natural discharge of the cerumen, removal through irrigation, manual removal, or a combination of these strategies (Ogunleye and Awobem, 2004; Schwartz *et al.*, 2017). Removal procedures are generally safe, and rare complications may include vertigo, otalgia, tympanic membrane perforation, and bruised external ear canals (Ogunleye and Awobem, 2004; Gabriel, 2015). Depending on the severity of the cerumen impaction, cerumen removal may reportedly improve air conduction hearing thresholds by 5–40 dB (Sharp *et al.*, 1990; Roeser, and Ballachanda, 1997; Schwartz *et al.*, 2017; WHO, 2021). Consequently, untreated cerumen impaction can lead to mild hearing loss or exacerbate pre-existing SNHL, underscoring the importance of timely and appropriate management.

In low-income community settings, where cerumen impaction is a prevalent concern, local practices for self-treatment often prevail, which might include unregulated use of home remedies or over-the-counter solutions. While culturally ingrained, these practices may overlook the effectiveness and cost implications of various medically approved methods (Loveman *et al.*, 2011). Typically, cerumen removal services are provided by audiologists, otolaryngologists, or other trained healthcare personnel, such as nurses in primary healthcare (PHC) facilities and community settings (Mulwafu *et al.*, 2016; Munro *et al.*, 2023). However, the scarcity of these professionals in low-income areas often leads to the neglect or absence of proper cerumen impaction treatment. This underscores the necessity for task-shifting strategies, where training community healthcare workers (CHWs) to manage common ear problems, like cerumen impaction, could significantly enhance access to care at a primary level (WHO, 2021). Such an approach not only mitigates the shortage of specialized healthcare providers but also ensures cost-effective and culturally sensitive management of ear health in these communities (Chadha *et al.*, 2018; Yousuf Hussein *et al.*, 2018; Orji *et al.*, 2020; WHO, 2021).

The World Report on Hearing (2021) acknowledges the importance of providing accessible and affordable hearing care services within communities. Decentralized and community-based hearing care services are a potential tool to provide services to individuals with hearing loss in LMICs (WHO, 2012; Mulwafu *et al.*, 2017; WHO, 2021; Frisby *et al.*, 2022a). Shifting audiological services to PHC facilities is a crucial step toward efficient identification and management of hearing loss in LMICs, enhancing early detection and intervention (WHO, 2012; Louw *et al.*, 2018b; Baum *et al.*, 2019; O’Donovan *et al.*, 2019; WHO, 2021; Frisby *et al.*, 2022b; WHO, 2023). Integrating innovative mobile health (mHealth) and telehealth technologies is a key recommendation to support this decentralization. These technologies, including remote hearing assessments and data management tools, facilitate broader reach and efficient service delivery. (Swanepoel *et al.*, 2010; Yousuf Hussein *et al.*, 2016; Van Wyk *et al.*, 2019; WHO, 2021; Frisby *et al.*, 2022a).

Mobile technologies demonstrate increasing promise for community-based hearing care, particularly in resource-limited settings. For example, digital application-based automated audiometry, equipped with transducer features to attenuate background noise, offers a practical solution for conducting quick and reliable hearing tests for both adults and children (Yousuf Hussein *et al.*, 2018; Dawood *et al.*, 2020; Swanepoel, 2023). These user-friendly technologies enable minimally trained non-specialist CHWs to operate automated audiometric equipment successfully, thus facilitating task-shifting in hearing care services (Bright *et al.*, 2019; Dawood *et al.*, 2020). Additionally, innovative tools such as artificial intelligence (AI)-assisted classification of ear status, utilizing smartphone video otoscopy, exemplify the advanced mHealth solutions available to support community-based hearing care. These tools provide simple yet effective triaging capabilities, enhancing early detection and management of ear conditions (Jayawardena *et al.*, 2020; Pendersen, 2020; Swanepoel, 2023). Importantly, the implementation of these technologies can be facilitated by CHWs who receive remote support and follow-up by audiologists (Yousuf Hussein *et al.*, 2018; Van Wyk *et al.*, 2019; Dawood *et al.*, 2020; Frisby *et al.*, 2022a).

The implementation of scalable, community-based service-delivery models, especially those led by CHWs supported by innovative technologies, has emerged as an important priority for enhancing access to hearing care in low-income environments (WHO, 2021; Frisby *et al.*, 2022a; Swanepoel, 2023). Addressing this need, this study explored adult hearing loss and cerumen management within a community setting, specifically employing mHealth technologies facilitated by CHWs. This approach bridges a notable gap in the current research concerning the clinical and cost-effectiveness of cerumen management in primary healthcare settings (Loveman *et al.*, 2011) and adopts a robust methodological framework to yield insightful data. Consequently, this study contributes to the growing body of evidence for innovative, community-based hearing care models and provides practical insights for implementing such models effectively in low-income settings.

## Methods

IRB approval was granted by the Faculty of Humanities Research and Ethics Committee of the University of Pretoria (Approval number HUM032/0523), and all participants had to provide informed consent before participating. This study was done in collaboration with the hearX Foundation and two non-governmental organizations (NGOs).

### Study design

A cross-sectional study design, following a predominantly quantitative, descriptive research approach, was used in this study. This study aimed to describe the prevalence and nature of hearing loss in a self-referred group of adults receiving hearing services through a community-based model. A secondary objective was to evaluate the effectiveness of community-based treatment for cerumen impaction.

### Participants

Participants were recruited through a partnership with the hearX Foundation at the Ikamva Labantu Community Centre in Khayelitsha and the Mbekweni Kuyasa Elderly Centre in Paarl, Western Cape, South Africa. Both facilities are located within low-income communities. Khayelitsha is one of the largest and highly populated residential townships within the City of Cape Town municipality, with 400 000 people reported to reside there more than 10 years ago (Statistics South Africa, 2011a). It is characterized by informal settlements alongside more formalized housing and is faced with multiple socio-economic challenges, including high unemployment rates, inadequate access to basic services such as healthcare and education, contributing to broader social disparities. Mbekweni is situated between Paarl and Wellington within the Drakenstein municipality, with over 30 000 residents (Statistics South Africa, 2011b). It is characterized by a mix of urban and rural elements, high unemployment rates, and low education levels.

The community-based NGOs (Ikamva Labantu and Mbekweni Kuyasa) aim to empower older community members through community-led projects. The responsible NGOs indicated that all members and their family members with concerns about their hearing could participate in the study.

Community members had to meet the following inclusion criteria to be considered participants: i) 18 years and older, ii) self-reported hearing difficulties, iii) community member at the Ikamva Labantu or Mbekweni Kuyasa community centres. Only participants who presented with cerumen impaction either unilaterally or bilaterally and were willing to undergo cerumen management procedures were considered for cerumen management.

The project was facilitated by two trained CHWs with over three years of experience, who were employed by the hearX Foundation and responsible for implementing the service-delivery model. Their training, conducted by a qualified audiologist (project manager), covered various hearing healthcare services, including hearing screening, hearing assessment, video otoscopy, digital automated audiometry, hearing aid fittings, and device care. Additionally, an onsite nurse was responsible for performing cerumen management procedures including offering sweet oil bottles, and removal through irrigation.

### Material and apparatus

Digital video otoscopy (hearScope™, hearX Group, Pretoria, South Africa) connected to a smartphone (Samsung Galaxy A3 smartphone) was conducted to visualize the ear canal and tympanic membrane. A beta version of the hearScope™ AI classification algorithm was used to provide an automated classification of the tympanic membrane image. Therefore, the CHW was not responsible for interpreting otoscopic results. Smartphone-based (Samsung Galaxy A3 smartphone) automated audiometry was conducted on an audiometry application (hearTest™, hearX Group, Pretoria, South Africa), coupled with headphones (Supra-aural Sennheiser HD 280 Pro headphones, Wedemark, Germany) to obtain hearing thresholds. The Sennheiser HD 280 Pro headphones were calibrated according to reference equivalent threshold sound pressure levels (RETSPL), adhering to equivalent threshold sound pressure levels approved for these headphones (Madsen and Margolis, 2014). Cerumen was irrigated from the ear canal with lukewarm water (OtoClear® Ear Wash Kit, Bionix Medical Technologies, Ohio, USA). Lighted curettes (Lighted Ear Curette™ with Magnification, Bionix, Medical Technologies, Ohio, USA) were also used to manually remove the cerumen if necessary.

### Procedures

The CHW administered a brief pre-testing questionnaire (Supplementary material I) in an interview format to gather information about the participant’s hearing abilities. The CHW then facilitated the hearing evaluation, which included video otoscopy and automated pure-tone audiometry. For any outer and/or middle ear defects (e.g., discharge, ear infection, perforated tympanic membrane, etc.) other than cerumen impaction found during AI-assisted video-otoscopic evaluation, the CHW made appropriate referrals. The AI classification beta version of the video otoscope classified findings into four main categories: i) Normal, representing a clear and healthy outer ear and tympanic membrane with no abnormalities detected; ii) Abnormal, indicating any structural anomalies of the outer/middle ear (any disorder of the outer or middle ear); iii) Cerumen impaction, any ear with occluding, impacted cerumen and iv) Unable to determine, which represents all otoscopic results that could not be classified into any of the other categories by the video otoscope due to factors such as low-resolution imaging of the ear canal, lack of training data (limited exposure to diverse sample ear canal images), and complex or atypical cases.

Air conduction pure-tone audiometry was then obtained for hearing thresholds at 500, 1000, 2000, 4000, 6000, and 8000 Hz bilaterally for the Ikamva Labantu participants, while frequencies 500, 1000, 2000, and 4000 Hz were tested for the Mbekweni Kuyasa participants. The Mbekweni Kuyasa participants were tested up to 4000 Hz for time efficiency purposes, while still obtaining significant hearing frequency information crucial for speech understanding. Initially, Ikamva Labantu was the only facility from which participants were to be recruited. However, during the course of the study, an opportunity arose to include additional participants from Mbekweni Kuyasa. This increase in sample size was anticipated to strengthen the study findings and enhance the representativeness of different demographics, which can be applied to a wider population.

The Ikamva Labantu participants were tested using a minimum output level of 20 dB at each frequency (to account for potential background noise interference), while 0 dB was used for the Mbekweni Kuyasa participants. The maximum output protocol was the same for both the community centre’s participants; 90 dB for frequencies 500 to 4000 Hz, 80 dB for 6000 Hz, and 70 dB for 8000 Hz. Participants had the choice of responding to pure tones by raising their hand, thus facilitated by the CHW, or to self-test, wherein the participant operated the digital audiometry smartphone themselves. Prior to the commencement of the pure-tone audiometric assessment, the CHW provided the participants with instructions for the test and a conditioning phase.

**Figure 1.**
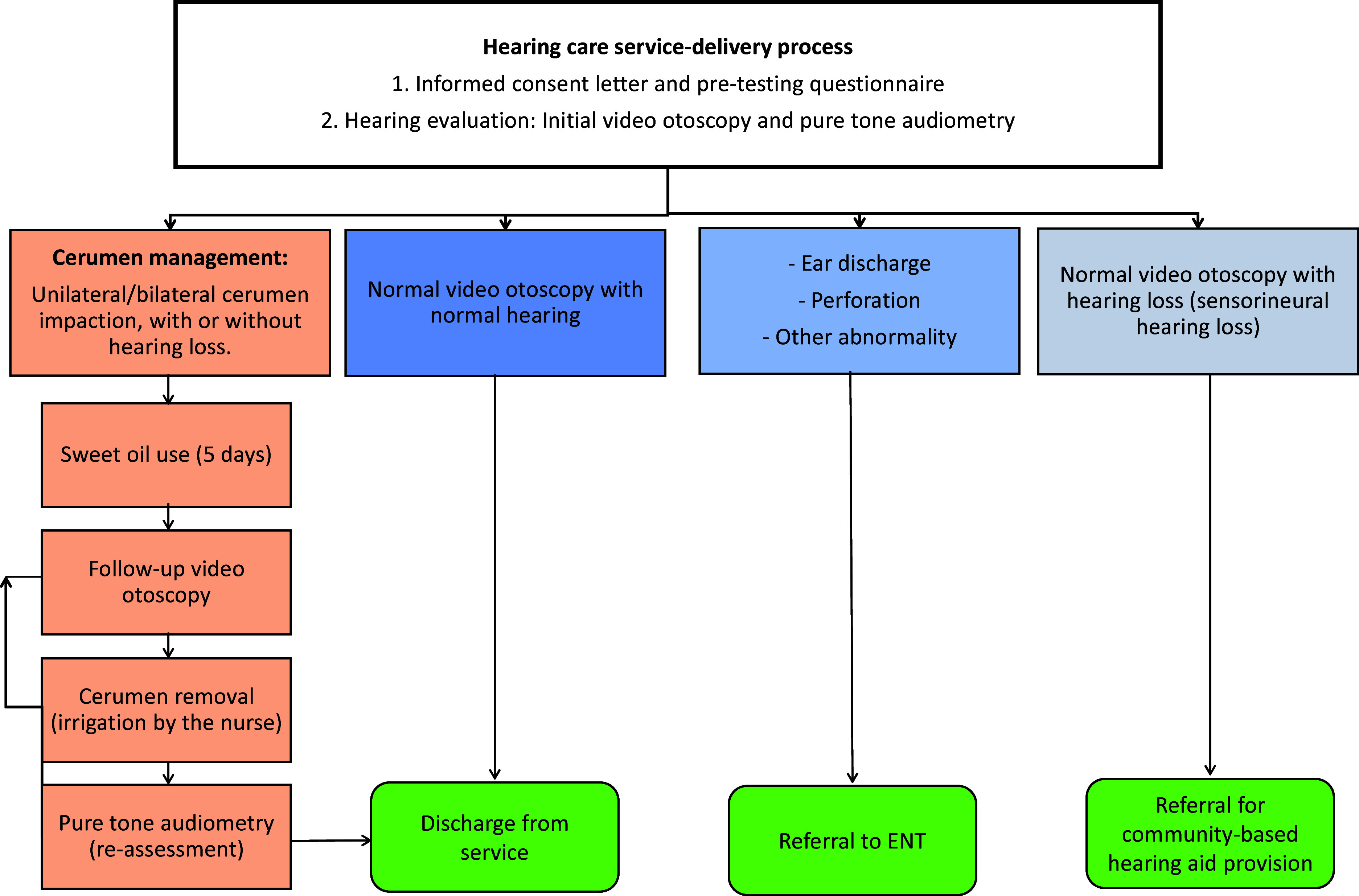
Community-based hearing healthcare service-delivery model.

Once a complete hearing evaluation was conducted, participants identified with cerumen impaction through the video otoscopy and AI classification were encouraged to undergo cerumen management. The cerumen management plan consisted of a cerumenolytic agent (sweet oil) used for a minimum of five days, followed by nurse-administered syringing or manual removal at the community centre. The participants with cerumen impaction were instructed to place 2–3 drops of the sweet oil into their ear(s) with impacted cerumen once a day for five days. This was to allow softening and natural discharge of the cerumen. Once the sweet oil had been administered for five days, the participant had to return to the centre for a follow-up appointment, where video otoscopy was conducted again. If the cerumen was still found to be impacted an onsite nurse subsequently removed the cerumen through irrigation or manual removal. If the cerumen was not soft enough for removal, the nurse recommended another week of sweet oil use. Follow-up video otoscopy and pure-tone audiometry re-assessment were conducted following cerumen removal. Data collection procedures are summarized below (Figure 1).

#### Classification of hearing loss

Hearing loss was classified according to the WHO (2023) classification system with minor adjustments. Normal hearing was defined as a four-frequency (500, 1000, 2000, and 4000 Hz) PTA of 20 dB based on the minimum testing level implemented. Mild hearing loss was defined as the four-frequency PTA between >20 and <35 dB HL.

Type of hearing loss was classified based on otoscopy and pure-tone audiometry results. Four groups were used, including sensorineural hearing loss (SNHL), conductive or mixed hearing loss, cerumen impaction with hearing loss, and undefined hearing loss. SNHL was classified based on normal video-otoscopic results with a PTA hearing loss. Normal video-otoscopic classification results were assumed to represent no obvious abnormalities in the external ear canal, tympanic membrane, or middle ear structures (Caroça *et al.*, 2017). Hearing loss with abnormal otoscopy was thus classified as either conductive or mixed hearing loss. If video otoscopy identified cerumen impaction, participants were classified as cerumen impaction with hearing loss. The undefined classification covers all those whose otoscopic results could not be classified by the video otoscope (unable to determine group) and those who did not have otoscopy results.

### Analysis

Analysis of statistical data was performed using the IBM SPSS version 28 for Windows. Mean and standard deviation (SD) values of participant ages and audiometry thresholds were determined. The otoscopic results were descriptively analysed using subcategories based on whether participants had the same or different otoscopic findings in both ears. We identified bilateral cases, where both ears of a participant showed the same result, and unilateral pairs, where each ear showed different results. For unilateral pairs, we matched the different otoscopic findings from each participant’s left and right ears. These pairs included normal and abnormal, normal and cerumen impaction, normal and undetermined, abnormal and cerumen impaction, and cerumen impaction and undetermined.

To evaluate the distribution of the hearing thresholds and pure-tone average (PTA) data, the Kolmogorov-Smirnov test of normality (Razali and Wah, 2011) and visual inspection of the normal Q-Q plots for hearing thresholds at each frequency and PTA were performed for each ear. The value for level of significance was accepted to be *P* < 0.05. A paired sample *t*-test was performed to examine left and right ear differences in audiometric data for all participants, and no overall significant difference was found between the left and right ears across frequencies 500–4000 Hz (*P* > 0.05). Therefore, the hearing status (hearing thresholds and PTA) data was pooled for the left and right ears (Coren and Hakstian, 1990). An independent sample *t*-test for gender difference could not be performed due to the skewed distribution of males (*n* = 41) compared to females (*n* = 186).

## Results

A total of 227 self-referred adults (81.9%; *n* = 186 females) aged between 43 and 102 years (mean 71.8 ± 8.6 SD) were assessed at the community centres. Video otoscopy and pure-tone audiometry results for all participants are presented below.

### Hearing evaluation

#### Initial video otoscopy

Video otoscopy was conducted for 98.7% (*n* = 224) of participants. Three participants did not receive an otoscopic examination. Reasons included technical issues with the system (*n* = 2) and difficulty understanding instructions (*n* = 1). Table 1 shows the initial video otoscopic results of all 224 participants and 448 ears.

**Table 1.** Initial video otoscopy AI classification results across ears (*n* = 448) and participants (*n* = 224)

Category	Ears % (*n*)	Participants % (*n*)
	*(n = 448)*	*(n = 224)*
Normal	57.9 (263)	46.3 (105)
Cerumen impaction	29.1 (132)	19.4 (44)
Unable to determine	10.4 (47)	7.0 (16)
Abnormal	1.3 (6)	0.4 (1)
Normal/Cerumen impaction	–	17.2 (39)
Normal/Unable to determine	–	4.8 (11)
Cerumen impaction/Unable to determine	–	1.8 (4)
Normal/Abnormal	–	1.3 (3)
Abnormal/Cerumen impaction	–	0.4 (1)

Across the total of 448 ears tested, based on the AI classification (hearScope Beta AI classification, hearX Group, South Africa) normal otoscopic results were the most common (57.9%). In contrast, abnormal classifications only occurred in 1.3% of ears. A combined 70.5% of participants (*n* = 158/224) had normal otoscopy in at least one ear, 39.3% (*n* = 88/224) had cerumen impaction in at least one ear, and 2.2% (*n* = 5/224) had abnormal otoscopy in at least one ear.

#### Degree of hearing loss

Of the 227 adults tested, 56.4% required assistance from the CHW during the hearing assessment and thus raised their hand as a response indication. Table 2 displays the mean hearing thresholds obtained for all participants.

**Table 2.** Average hearing thresholds (dB HL) for all participants (*n* = 227)

		Frequency (Hz)					
		500	1000	2000	4000	6000	8000
Left ears	*n*	226	227	227	227	190	191
	Mean (SD)	43.5 (20.7)	41.4 (21.4)	44.9 (24.0)	47.4 (22.5)	50.4 (21.9)	50.3 (19.3)
Right ears	n	227	227	227	227	190	191
	Mean (SD)	43.5 (19.5)	41.3 (20.5)	42.2 (21.9)	45.3 (22.8)	50.8 (21.6)	51.9 (18.5)
Ears combined	*n*	453	454	454	454	380	382
	Mean (SD)	43.5 (20.1)	41.3 (20.9)	43.6 (22.9)	46.3 (22.7)	50.6 (21.8)	51.1 (18.7)

Mean hearing thresholds (dB) = Decibel(s); Frequency (Hz) = Hertz; SD = Standard Deviation.

**Figure 2.**
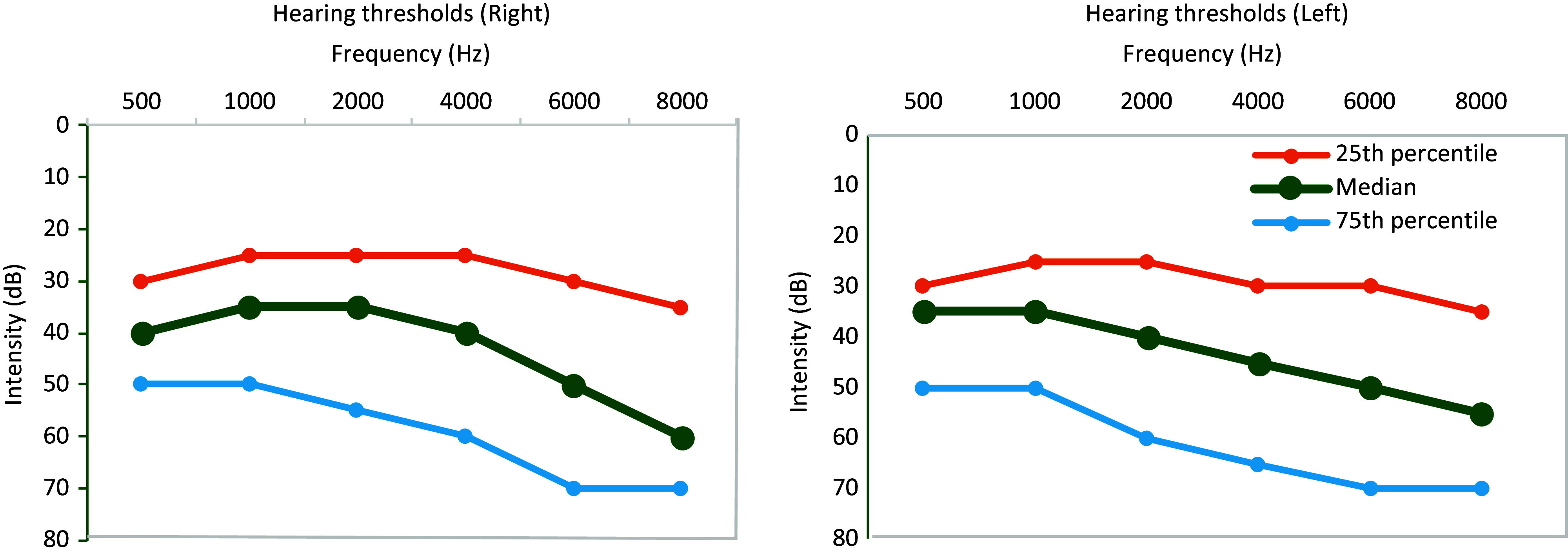
Distribution of hearing thresholds for participants’ left and right ears.

Generally, the hearing thresholds sloped towards the higher frequencies (Figure 2; Table 2). The PTA of the combined ears had a mean hearing threshold of 43.7 dB (±19.3 SD) and a minimum and maximum range of 15–90 dB. Almost all (97.8%) participants presented with some degree of hearing loss, 95.2% (*n* = 216) of which had bilateral hearing loss, while 2.2% (*n* = 5) had normal bilateral hearing, and none had unilateral hearing loss (Table 3). Mild hearing loss was the most common degree of loss (45.8%; Table 3).

**Table 3.** Grading of hearing loss for all participants assessed (*n* = 227)

^*^Degree of hearing loss	Male *(n = 41)* % (*n*)	Female *(n = 186)* % (*n*)	All participants *(n = 227)* % (*n*)
Normal hearing *(0–20 dB)*	4.9 (2)	4.8 (9)	4.8 (11)
Mild hearing loss *(21–34 dB)*	43.9 (18)	46.2 (86)	45.8 (104)
Moderate hearing loss *(35–49 dB)*	22.0 (9)	26.9 (50)	26.0 (59)
Moderately severe hearing loss *(50–64 dB)*	17.1 (7)	10.2 (19)	11.5 (26)
Severe hearing loss *(65–79 dB)*	4.9 (2)	8.1 (15)	7.5 (17)
Profound hearing loss *(80–94 dB)*	7.3 (3)	3.8 (7)	4.4 (10)

* Degree of hearing loss: Graded according to each participant’s better ear four-frequency PTA (WHO, 2023).

#### Type of hearing loss

Across 454 ears, SNHL was the most common (*n* = 251; 55.3%), followed by cerumen impaction with hearing loss (*n* = 129; 28.4%), undefined hearing loss (*n* = 53; 11.7%), normal hearing (*n* = 15; 2.6%), three of which were with cerumen impaction, and conductive or mixed hearing loss (*n* = 6; 1.3%).

### Participants with cerumen impaction

Of the 227 total participants in this study, 88 (39.3%) were identified with cerumen impaction in at least one ear and were all offered sweet oil by the CHW. Of these, 17 (19.3%) participants were not willing to take part in the cerumen management procedures. Furthermore, only 54 (76.1%) out of 71 participants who received sweet oil returned for a follow-up session. Several factors led to non-participation in the cerumen removal and follow-up assessment, including discontinuation of community centre membership (*n* = 2), loss of contact (*n* = 3), unavailability (*n* = 4), illness (*n* = 2), referral for further evaluation (*n* = 4), and refusal to undergo cerumen removal procedures due to denial of ear and/or hearing problems (*n* = 1), and other non-disclosed personal reasons (*n* = 1).

#### Follow-up video otoscopy

Video otoscopy was successfully done for 47 (87%) of the 54 follow-up participants. Video otoscopy could not be done for 7 participants due to technical issues with the video otoscope. Table 4 displays the follow-up otoscopy results after the cerumen removal.

**Table 4.** Follow-up video otoscopy AI classification results across ears (*n* = 94) and participants (*n* = 47) post-cerumen removal

Category	Ears % (*n*)	Participants % (*n*)
	*(n = 94)*	*(n = 47)*
Normal	50.9 (55)	38.9 (21)
Cerumen impaction	31.5 (34)	18.5 (10)
Unable to determine	2.8 (3)	1.9 (1)
Abnormal	1.9 (2)	–
Normal/Cerumen impaction	–	22.2 (12)
Cerumen impaction/Unable to determine	–	1.9 (1)
Normal/Abnormal	–	1.9 (1)
Abnormal/Cerumen impaction	–	1.9 (1)

Post-cerumen removal, more than half (*n* = 55; 50.9%) of the affected ears presented with normal video otoscopy classifications.

#### Follow-up hearing loss classification

Within the total 108 ears initially presenting with cerumen impaction (pre-cerumen removal), 105 (97.2%) ears presented with hearing loss, while 3 (2.8%) had normal hearing. Post-cerumen removal, 106 (98.1%) ears presented with hearing loss, while 2 (1.9%) had normal hearing. Of those with hearing loss post-cerumen removal (106 ears), 54 (50.9%) presented with SNHL, 34 (32.1%) cerumen impaction with hearing loss, 16 (15.1%) undefined hearing loss, and 2 (1.9%) conductive or mixed hearing loss.

The mean difference between the pre- and post-cerumen removal PTA was 3.9 (±20.4 SD) for the left ears (*n* = 54), 1.6 (±20.7 SD) for the right ears (*n* = 54), and 2.7 (±20.5 SD) for all ears combined (*n* = 108). Of the total participants, 29 (53.7%) and 27 (50%) had PTA improvement ranging from 1.2 to 68.8 dB and 1.2 to 67.5 dB in the left and right ears, respectively post-cerumen removal. There were 14 left and 12 right ears with an improvement of 10 dB or more. Two left and right ears each had a ≥ 40 dB PTA improvement post-cerumen removal. All pre-cerumen removal mean PTA values were slightly higher than post-cerumen removal mean PTA values; however, there was no statistically significant difference (*P* > 0.05) between them. A significant difference was observed in the mean hearing thresholds at 2000 Hz pre- and post-cerumen removal for the left ear and for both ears combined (*P* < 0.05). However, this significant change was not observed at any other tested frequencies (500–8000 Hz) for the left ear, right ear, or when both ears were considered together (*P* > 0.05). The mean threshold differences and their respective *P*-values are displayed in Table 5.

**Table 5.** Comparison of hearing thresholds (dB) before and after cerumen removal in participants (*n* = 54)

		Frequency					
		500	1000	2000	4000	6000	8000
Left ears	*n*	54	54	54	54	41	44
	Ave dB improvement (SD)	1.6 (23.8)	2.0 (24.7)	7.6 (27.7)	4.5 (22.9)	3.5 (18.3)	4.1 (13.9)
	*P*-value	0.63	0.55	0.05*	0.15	0.22	0.06
Right ears	*n*	54	54	54	54	41	44
	Ave dB improvement (SD)	–0.1 (23.7)	–0.3 (22.8)	4.5 (25.0)	2.0 (24.6)	–0.1 (25.9)	0.9 (13.1)
	*P*-value	0.98	0.93	0.19	0.55	0.99	0.65
All ears	*n*	108	108	108	108	82	88
	Ave dB improvement (SD)	0.7 (23.7)	0.9 (23.7)	6.1 (26.3)	3.3 (23.7)	1.7 (22.4)	2.5 (13.5)
	*P*-value	0.75	0.70	0.02*	0.15	0.49	0.09

Average dB improvement (improvement in hearing from pre- to post-cerumen removal); dB = Decibel(s); Hz = Hertz; SD = Standard Deviation.

## Discussion

This study describes the prevalence and nature of hearing loss in a self-referred adult cohort (average age 71.8 years) within a low-income community while evaluating the effectiveness of a simple community-based cerumen management protocol. While the majority of participants had normal otoscopic findings, a high prevalence of cerumen impaction was observed, alongside a spectrum of hearing loss severities, with SNHL being predominant. This study is the first to assess the outcomes of a community-based treatment approach for cerumen impaction, using a combination of digital otoscopy and AI classification to support CHW-led interventions. Successful management of cerumen impaction in communities emphasizes the importance of cerumen management in primary ear health strategies, particularly in low-income settings.

The incidence of hearing loss was almost universal (97.8%) in this sample of self-referred older adults. Prevalence rates in the current study are higher than those in both low- and high-income settings that reported varying rates of between 52% and 82% in older adults (>60 years) (Olaosun *et al.*, 2013; Homans *et al.*, 2017; WHO, 2021; Li *et al.*, 2023; Reed *et al.*, 2023). The high prevalence in the present study is most likely due to the inclusion criteria, which required participants to have self-reported hearing difficulties. Studies have shown that adults who self-report hearing loss present with significantly higher rates of confirmed hearing loss (Sindhusake *et al.*, 2001; Torre *et al.*, 2006; Louw *et al.*, 2018b). The limited number of participants with normal hearing in the self-referred group emphasizes the value of self-reported hearing difficulties as an initial screening tool in resource-constrained settings (Torre *et al.*, 2006; Louw *et al.*, 2018b). Furthermore, the unique geographic and socio-economic characteristics of the low-income, LMIC-based community also potentially contributed to the elevated rates in this study due to environmental risks (Cunningham and Tucci, 2017; WHO, 2021).

The severity of hearing loss in our study ranged from mild to profound, with mild hearing loss being the most common, affecting 45.8% of participants. This contrasts with findings from other studies in Africa, where moderate to moderately severe hearing loss was more prevalent among adults over 55 years (Olaosun *et al.*, 2013; Abdel-Hamid *et al.*, 2007). However, this is in line with the global trend reported by WHO (2021), which indicates that mild hearing loss is most prevalent worldwide. In Africa, the age-standardized prevalence of hearing loss of moderate or greater severity was reported to be 5.4%, which is greater than in other regions including Europe and America (3.5 and 4.7% respectively) (GBD, 2021). In our study, moderate or more severe hearing loss was observed in 51.9% of the cases, which is lower than the 67% reported in a Nigerian study (Olaosun *et al.*, 2013) of patients aged 65 and older and higher than the 30% observed in a Dutch study (Homans *et al.*, 2017) of adults aged 65 and over. However, it is also important to note that the higher rates of moderate to severe hearing losses reported in African studies, including ours, likely reflect a broader trend within low-income settings. As opposed to other regions, the higher prevalence and severity of hearing loss in Africa is attributable to a range of environmental, socio-economic, and healthcare access factors.

While SNHL was the predominant type of hearing loss observed in our study (55.3%), a relatively small percentage of participants (1.6%) had ear conditions such as conductive or mixed hearing loss requiring medical attention. This aligns with findings from a Nigerian hospital-based study (Ologe *et al.*, 2005), which reported a conductive or mixed hearing loss prevalence of 0.3–1.3% in adults aged 60 and above. Consistently, research studies from both African and European countries have indicated a low prevalence of middle ear-related hearing loss (Mulwafu *et al.*, 2016; Louw *et al.*, 2018a; Hoff *et al.*, 2020), suggesting that middle ear pathologies contribute minimally to overall hearing loss prevalence in adults. Conversely, cerumen impaction was a significant issue, affecting 28.4% of our study participants. Similarly, Ologe *et al.* (2005) found a 34.4% prevalence of cerumen impaction among adults over 60, and Lewis-Cullinan & Janken (1990) reported a 35% rate in adults over 65. These figures correspond closely with our findings, where cerumen impaction affected 39.3% of participants in at least one ear. Studies have demonstrated that cerumen impaction can be found in up to 57% of older adults (McCarter *et al.*, 2007; WHO, 2021). Lower rates of excessive cerumen were reported (18.6–22.4%) from an American national survey data set in those aged >70 years (Humes, 2024). The variation in these results may stem from the utilization of different otoscopy categorizations. Humes (2024) distinguished between cerumen impaction and excessive cerumen. In contrast, in the present study, cerumen impaction was treated as inclusive of all observations of significant cerumen identified by the video otoscope AI, irrespective of whether it constituted excessive or obstructing cerumen.

The subgroup of participants with cerumen impaction demonstrated the effectiveness of community-based cerumen management, employing sweet oil and irrigation techniques. This approach, validated by previous studies (Ogunleye and Awobem, 2004; Loveman *et al.*, 2011; Gabriel, 2015; Munro *et al.*, 2023), highlights the feasibility and efficacy of simple, accessible treatments within a primary ear and hearing care framework. Furthermore, task-shifting, as advocated by the WHO and supported by various studies (Yousuf Hussein *et al.*, 2016; Bright *et al.*, 2019; Van Wyk *et al.*, 2019; WHO, 2021; Frisby *et al.*, 2022a), represents a cost-effective solution to the treatment of cerumen impaction, particularly in PHC settings. Non-specialist healthcare workers, alongside nurses, play a crucial role in the PHC sector for successfully assessing and treating ear and hearing problems such as cerumen impaction when adequately trained (Mulwafu *et al.*, 2016; Dawood *et al.*, 2020; Frisby *et al.*, 2022a; Munro *et al.*, 2023). This is particularly significant as studies have demonstrated an unmet need for cerumen management, predominantly in the PHC sector (Gabriel, 2015; Munro *et al.*, 2023).

The improvement of hearing thresholds post-cerumen removal varied among individuals, with more than half the participants presenting with some improvement. Our improvement rates were lower compared to a study by Lewis-Cullinan & Janken (1990), which reported hearing improvements in 75% of hospitalized participants post-cerumen removal. This difference could be attributed to the distinct study methodologies, including our employment of AI for assessments and the involvement of CHWs and nurses, in contrast to the specialists in the comparison study. Nonetheless, a subset of our participants experienced significant auditory improvement. Post-cerumen removal, 14 left and 12 right ears had PTA improvements of at least 10 dB, with a minority (two left and two right ears) exceeding 40 dB. While cerumen removal improved hearing thresholds for some individuals, the improvement across the entire the subgroup was minimal. This could be partly explained by the varying severity of cerumen impaction among participants, ranging from partial to full blockage. The AI-integrated video otoscope used in this study was not designed to distinguish between degrees of impaction severity, but rather the broad otoscopy categories.

A number of limitations may have influenced the results and interpretation of this study’s findings. The female-to-male ratio (more female participants) presents potential constraints in generalising findings across both genders, particularly since males are more prone to developing hearing loss than females (Nam *et al.*, 2024). The implications of the selection bias in this study require careful consideration when generalising the findings to broader populations, as the estimates may not accurately reflect the true extent of hearing loss in the wider community. Although minimal, the exclusion of individuals without video otoscopy data (*n* = 10) for both pre- and post-cerumen removal may have affected the sample’s representativeness, potentially leading to an underrepresentation of certain categories. The inability to distinguish between various cerumen impaction severities may have influenced the ability to fully assess the impact of cerumen removal on hearing thresholds.

## Conclusion

The persistent need for enhanced hearing care services in the PHC sector, particularly in low-income and elderly populations, is underscored by the study’s findings. The novel community-based cerumen management plan was effective in restoring the integrity of the outer ear canal, even without complete restoration of hearing thresholds for some participants. Task-shifting, involving CHWs and nurses, utilizing mHealth technologies proved effective in identifying and addressing ear and hearing problems through a cost-effective service-delivery model. The integration of community resources and task-shifting strategies has the potential to enhance ear and hearing care accessibility in resource-constrained settings.

## Supporting information

Manganye et al. supplementary material Manganye et al. supplementary material

## Supplementary material

The supplementary material for this article can be found at https://doi.org/10.1017/S1463423625000246
