# Supplementary material for: Hearing loss characteristics and cerumen management efficacy in low-income South African communities: a cross-sectional study
Source: Prim Health Care Res Dev. 2025 Mar 10;26:e27. doi: 10.1017/S1463423625000246 (PMC11955542; doi:10.1017/S1463423625000246)
Supplement: Manganye et al. supplementary material [file S1463423625000246sup001.pdf]

PRE-TESTING QUESTIONNAAIRE FOR PARTICIPANTS

**Participant number:**	
**Date completed:**	
**Field worker number:**	
**Participant contact number:**	
**Participant contact number: (Alternative)**	

Pretesting questionnaire (for all participants)

1. Which of the following cases (sentences/statements) best describe *how you feel about your hearing* at the moment?

	I do not think I have a hearing problem.
	I think I have a hearing problem. However, I am not yet ready to take any action to solve the problem, but I might do so in the future.
	I know I have a hearing problem, and I intend to take action to solve it soon.
	I know I have a hearing problem, and I am here to take action to solve it now.
	None of these symptoms.

2. For how long have you had a problem with your hearing? (Ask the participant to specify, i.e., 6 months, 3 years, or longer than 10 years)

____

____

____

3. Do you have any of the following symptoms?

	Sudden hearing loss: You woke up one morning and suddenly could not hear as well as you could the day before
	You hear a ringing (shh shh sounds) in your ears that is very loud and sounds like a heartbeat.
	Hearing loss in one ear only.
	You often feel dizzy.
	None of these symptoms.

4. Do you or someone in your home have a phone that can receive WhatsApp and/or SMSs?

	WhatsApp
	SMS
	No

If SMS or WhatsApp who has a phone

	Participant themselves
	Household member

If only a household member has a phone, who is this household member? (e.g. child, sister, uncle etc.) What is their contact number? ____

5. How comfortable are you in using a phone?

	I can receive phone calls.
	I know how to make and receive calls.
	I can make and receive calls and send and receive text messages via SMS.
	I can make and receive calls and send and receive text messages via WhatsApp.
	I need someone at home to help me use the phone.

6. Have you had your hearing tested before?

	Yes
	No
	Unsure

7. If yes, can you tell us where and what the results were?

____

____

____

8. Do you have a hearing aid?

	Yes
	No

If yes, do you use your hearing aid? Can you tell us why not if you do not use your hearing aid?

____

9. Have you had an ear infection or discharge from the ears in the last two years?

	Yes
	No
	Unsure

If yes, did you seek treatment? Was it a once-off problem or did the infection come back a few times?
